# From Tradition to Translation: A Critical Appraisal of *Bacopa monnieri* for Neuroprotection from Preclinical and Clinical Perspectives and Challenges in Utilization

**DOI:** 10.3390/ijms27125488

**Published:** 2026-06-17

**Authors:** Abosede Temitope Olajide, Sasithon Aunsorn, Samuel Abiodun Kehinde, Thammarat Kaewmanee, Sasitorn Chusri

**Affiliations:** 1 Biomedical Technology Research Group for Vulnerable Populations and School of Health Science, Mae Fah Luang University, Chiang Rai 57100, Thailand; abosedeolajide3@gmail.com (A.T.O.); samuelkehinde0707@gmail.com (S.A.K.); 2 Department of Thai Traditional Medicine, Faculty of Allied Health Sciences, Nakhon Ratchasima College, Nakhon Ratchasima 30000, Thailand; 6551811005@lamduan.mfu.ac.th; 3 Biochemical/EnTox Laboratory, Faculty of Basic Medical Sciences, Ajayi Crowther University, Oyo 211001, Nigeria; 4 Department of Food Science and Nutrition, Faculty of Science and Technology, Prince of Songkla University, Pattani 94000, Thailand; thammarat.k@psu.ac.th

**Keywords:** Alzheimer’s disease, *Bacopa monnieri*, bacosides, cognitive enhancement, dementia

## Abstract

Dementia, and more specifically Alzheimer’s disease (AD), is a progressive neurodegenerative disorder that has become a growing health menace in the world with an escalation in incidence as well as enormous social and economic consequences. Existing pharmacological treatment including cholinesterase inhibitors and N-methyl-D-aspartate (NMDA) receptor antagonists are not very effective in reducing the symptoms and fail to prevent the disease process. The non-pharmacological treatment interventions such as diet, exercise and cognitive training have supportive effects and cannot be used as standalone treatments. Therapeutic gap has resulted in increased interest in complementary and alternative therapies, especially that of pleiotropic action of herbal medicines. *Bacopa monnieri* (BM) is an Ayurvedic herb that has historically been used to treat memory enhancement and now has both preclinical and clinical evidence supporting its ability to modulate neurotransmission, reduce oxidative stress and suppress neuroinflammation. However, such difficulties as low bioavailability, instability of the environmental factors, and variations in formulations restrict its clinical applicability. New technologies with a lot of potential such as microencapsulation technology can provide the solution to this problem by increasing stability, solubility, and targeted delivery of compounds that will increase treatment efficacy. This narrative review is a synthesis of the existing information on the pathogenesis of dementia, therapeutic approaches, and the effectiveness of BM as a complementary intervention. It points out links between traditional medicine and modern neuroscience, strengths and limitations of on-going evidence, gaps that need further research, such as long-term clinical trials, standardized formulations, and discovery of the role of BM in the gut–brain axis. BM is a prime example of how herbal medicines can be used as a complement to conventional treatment and play a role in multi-modal approaches aimed at reducing the cognitive impairment associated with dementia.

## 1. Introduction

Dementia can be called one of the most acute health issues in the world throughout the 21st century. It is marked by a gradual deterioration of memory, reasoning, and cognitive abilities and has over 57 million affected people in the global population and almost 10 million new cases each year [1]. The most common subtype, comprising 60–80% of cases, and being both cognitively impairing and neuropsychiatric (depression, apathy, and aggression) is known as Alzheimer’s disease (AD) [2]. Dementia is known to be especially severe in the Asia–Pacific region, where population aging and risk factors associated with lifestyles intersect to form an increasing prevalence rate [3]. In addition to clinical manifestation, dementia has significant economic and social burdens, overloading the healthcare system and reducing the quality of life of both patients and caregivers [4].

Cure types are yet to be found, despite decades of biomedical inquiry. Existing pharmacological treatment, such as cholinesterase inhibitors and N-methyl-D-aspartate (NMDA) receptor antagonists, offer limited symptomatic benefits but cannot prevent neurodegeneration [5]. The non-pharmacological treatment of dementia (diet, exercise, and cognitive training) has supportive effects but cannot handle the multifaceted pathophysiology of the disease [6]. This therapeutic gap has created an increasing interest in complementary and alternative therapies, especially herbal medicines high in phytochemicals with neuroprotective and anti-inflammatory effects [7,8].

One such herb is *Bacopa monnieri* (BM) which is a traditional Ayurvedic herb and has received a lot of focus due to its memory-enhancing and neuroprotective properties. Preclinical and clinical trials indicate that BM can modulate neurotransmitter activity, decreasing oxidative stress, and neuroinflammatory mediators [9,10]. Nevertheless, it has limitations in its therapeutic application due to poor bioavailability and environmental instability [11]. New technologies such as microencapsulation have potential solutions, enhancing the stability of compounds, their solubility, and targeted delivery [12]. It is against this background that the current review summarizes existing evidence on the pathogenesis of dementia, therapeutic interventions, and the applicability of BM as a complementary intervention in neurodegenerative diseases.

## 2. Prevalence and Healthcare Burden Related to Dementia

**Figure 1:**
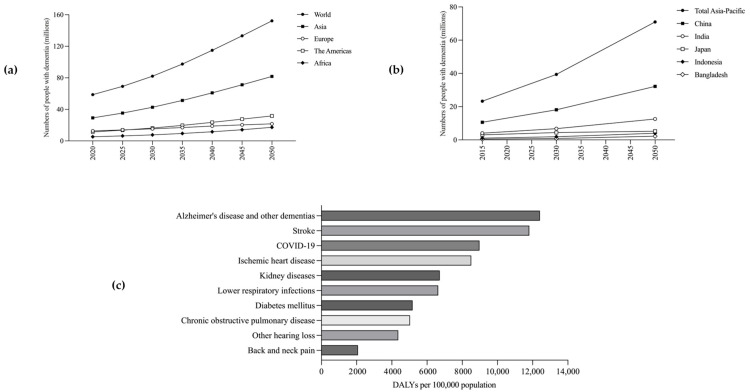
(**a**) The estimated global number of people with dementia (Adapted from Alzheimer’s Disease International, 2020). (**b**) The estimated number of people with dementia in the Asia–Pacific region dementia (Adapted from Alzheimer’s Disease International, 2020). (**c**) The cause of DALYs rate in the Thai elderly aged 85 and above (Adapted from World Health Organization, 2020).

AD is the most common type of neurodegenerative dementia and is experienced by about 5–6% of people over 65 and almost 30% of those over 85 years of age. About 5% of cases occur before age 65 (early onset AD). The disease has a characteristic progressive memory loss with cognitive deterioration and a median survival time of 10–12 years post-diagnosis [13,14]. According to the World Health Organization (WHO), in 2020, there were an estimated 59 million people living with dementia worldwide and this number is projected to grow to 82 million by 2030 and 152 million by 2050. Asia will have 82 million cases by mid-century, the Americas 32 million, Europe 21 million, and Africa 17 million [15] (Figure 1a). AD is currently the seventh leading cause of death and estimated to cause 1.62 million deaths worldwide in 2019 (1.06 million deaths in women and 0.56 million deaths in men) [16,17].

The cost to the economy of AD is just as enormous. The estimated total payments for health and long-term care for older adults (65+ years old) living with dementia reached $345 billion in 2023 and are projected to reach $465 billion in 2030, $763 billion in 2040, and more than $1 trillion by 2050 [18,19]. The individual-level annual care costs differ significantly depending on the severity of the disease as follows: $14,675 for mild AD, $19,975 for moderate AD, and $29,708 for severe AD (based on Mini-Mental State Examination [MMSE] scores) [20]. This spending includes activities that maintain quality of life, maximize functioning and promote safe cognitive, behavioral and social functioning.

The socioeconomic burden of AD and related dementias is very high in the Asia–Pacific region. The Alzheimer’s Disease International (ADI) 2014 report estimated that 23 million people in 2015 were affected by dementia in the region; that figure would rise to 39 million by 2030 and 71 million by 2050. The national burden is largest in China (32 million cases), India (13 million cases), and Japan (five million cases) (Figure 1b). In the region, the total expenditure on medical and social care for people with dementia amounted to $185 billion in 2015 and is projected to grow massively in countries with high aging rates and rapid urbanization, such as China, India, Japan, and Australia, where aging and urbanization are affecting the outcomes of medical and social care for people with dementia [3] (Table 1).

The prevalence of dementia in Thailand is between 2.35% and 3.1%, and the most common etiology in Thailand is AD [21]. An estimated 670,047 Thais lived with dementia in 2019, which is expected to rise to over 2.39 million in 2050 [22]. In the same year, there were 16,278 deaths from AD and other dementias, or 1.86% of all-cause mortality [23]. For those aged 85 years and older, the highest incidence of DALYs was due to dementia (12,409.15 per 100,000 population [24]) (Figure 1c). The costs of dementia in the national picture in 2015 were estimated at $1.8 billion, including $89 of direct medical costs, $721 in non-medical costs, and $854 in informal care costs per person [3] (Table 1).

## 3. Pathogenesis of Dementia

**Figure 2:**
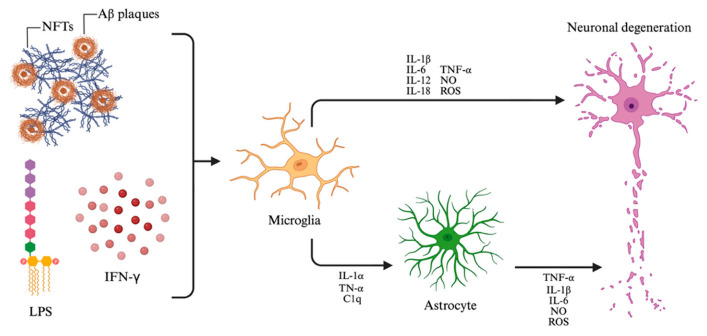
Neuropathological features of Alzheimer’s disease and dementia (Created in BioRender. Chusri, S. (2026) https://BioRender.com/gbsagq5 (accessed on 8 June 2026)).

The pathogenesis of dementia, especially AD, is multifactorial and complex, with a complex interaction between protein aggregation, oxidative stress, neuroinflammation and the dysregulation of the gut–brain axis (Figure 2). The amyloid hypothesis has been predominant in the study of dementia, and it states that the extracellular amyloid-beta (A2) peptides lead to the onset of a cascade of neurodegenerative events. The aggregation of A2 disrupts the synaptic signaling, activates kinases that hyperphosphorylate tau proteins, and forms neurofibrillary tangles (NFTs), which impair the axonal transport and subsequently cause neuronal apoptosis [24,25]. Although this model has informed therapeutic development, its weaknesses are becoming more apparent, with anti-amyloid interventions showing inconsistent clinical effects, leading to the demand to expand to multifactorial model.

Neuroinflammation has now become an important and mechanistically intricate contributor to the pathogenesis of dementia. Amyloid-beta species (ABS) bind to a set of pattern recognition receptors (PRRs) on microglia, such as toll-like receptors 2 and 4 (TLR2/4) in complex with the co-receptor cluster of differentiation 14 (CD14), the receptor for advanced glycation end-products (RAGEs), scavenger receptor cluster of differentiation 36 (CD36), and the C-type lectin Dectin-1. This binding to the receptor leads to the activation of downstream transcription factors, primarily nuclear factor kappa-light-chain-enhancer of activated B cells (NF-κB), which then activates the assembly of the inflammasome complex. The TLR2 and TLR4 signaling are both mediated by the myeloid differentiation primary response protein 88 (MyD88)-dependent pathway that activates NF-κB and activator protein 1 (AP-1), thereby enhancing the expression of pro-inflammatory mediators, chemokines and co-stimulatory molecules. At the same time, amyloid-beta (Aβ) activates NF-κB, which is the second signal to activate the NOD-like receptor family pyrin domain-containing 3 (NLRP3) inflammasome, while leakage of cathepsin B from the disrupted phagolysosomes or mitochondrial production of reactive oxygen species (ROS) serves as the second signal. NLRP3 assembly, which involves the complex of NLRP3, apoptosis-associated speck-like protein containing a caspase recruitment domain (ASC), and caspase-1, leads to the proteolytic maturation and secretion of interleukin-1-beta (IL-1β) and interleukin-18 (IL-18), as well as cleavage of Gasdermin D and formation of pores in the membrane, which cause pyroptosis. Activated M1-polarized microglia also signal through MAPK pathways, promoting the secretion of interleukin-6 (IL-6), interleukin-12 (IL-12), IL-1β, C-C motif chemokine ligand 2 (CCL2), and TNF-α and upregulation of costimulatory surface markers such as CD36, cluster of differentiation 11b (CD11b), and major histocompatibility complex class II (MHC-II) [26,27]. This cytokine environment is multi-level neurotoxic. Hyper-pression of IL-1β and tumor necrosis factor alpha (TNF-α) leads to a loss of synapses, partially via upregulation of prostaglandin E2. At the receptor level, inflammatory cytokines like TNF-α and IL-1β cause NMDA receptor overactivation, resulting in excessive Ca^2+^ influx, oxidative stress, activation of pro-apoptotic signaling through nuclear factor kappa B subunit 65 (p65) and nuclear factor kappa B subunit 53 (p53), and ultimately, synaptic dysfunction and neuronal degeneration. TNF-α also further compromises mitochondrial integrity by activating caspase-8 via binding to its receptor, tumor necrosis factor receptor 1 (TNFR1), which leads to the loss of mitochondrial membrane potential, release of cytochrome C, and subsequent disruption of the electron transport chain, resulting in decreased basal respiration, adenosine triphosphate (ATP) production, and maximal respiratory capacity. In this situation, reactive oxygen species (ROS) induce activation of NLRP3 via oxidation of potassium channels and intracellular calcium imbalance, creating a positive feedback loop of inflammation. The pro-amyloidogenic processing effects of IL-1β are due to activation of the c-Jun N-terminal kinase (JNK) pathway that facilitates amyloid accumulation and the mitogen-activated protein kinase (MAPK) pathway that induces neurofibrillary tangle formation, while IL-18 is involved in the upregulation of amyloid precursor protein (APP) and beta-site amyloid precursor protein cleaving enzyme 1 (BACE1) expression, thus increasing amyloidogenic processing [28,29].

Mechanistically, both IL-12 and IL-18 are closely involved in the neuroinflammatory network in AD. The IL-12/IL-23 pathway shares a common subunit of nuclear factor kappa B subunit 40 (p40) and is an important inflammatory pathway in the pathogenesis of AD, and genetic or pharmacological blockade of p40 significantly diminishes the AD-related pathology in transgenic amyloidosis models. IL-18 is an important mediator of neuroinflammation and neurodegeneration through pyroptotic cell-death pathways upon activation of the inflammasome, being constitutively expressed by cells resident in the brain. A major hallmark of activation of the NLRP3 signaling pathway, namely, the IL-18 signaling cascade, has been proposed as a central pathway involved in the AD continuum and is associated with tau pathology and synaptic degeneration. Microglial cells release interleukin-1-alpha (IL-1α), TNF-α, and complement component 1q (C1q), which then convert the astrocytes into a reactive A1 state that further perpetuates and escalates inflammation. This bidirectional glial crosstalk is essential to maintain the chronic neuro-inflammatory environment that triggers the loss of synapses, failure of mitochondria, excitotoxicity, and progressive neurodegeneration typical of AD [26,27,28,29].

Recent studies emphasize the gut–brain axis as a new aspect of the pathogenesis of dementia. Dysbiosis of the gut impairs the intestinal and blood–brain barrier integrity, permitting the entry of microbial metabolites like lipopolysaccharide (LPS) in the systemic circulation. LPS interacts with TLR4 (toll-like receptor) on microglia, triggering nuclear factor kappa-light-chain-enhancer of activated B cells (NF-kB) signaling and cytokine release [30]. The increased LPS in blood and brain tissue of AD patients has been associated with chronic endotoxemia causing cognitive impairment and small vessel disease [31,32]. Dementia-like pathology is reproduced in experimental models using LPS-induced neuroinflammation, which may be useful in understanding the role of inflammatory factors in cognitive impairment [33].

A combination of these processes, aggregation of proteins, oxidative stress, neuroinflammation, and gut dysbiosis exemplifies the complexity of the pathogenesis of dementia. It further discusses the reasons why single-target therapies have been unable to yield significant clinical improvement. There is an emerging agreement in favor of multi-targeted treatments that tackle the interrelated pathways that mediate neurodegeneration. This awareness preconditions the discussion of the interventions such as BM, which display pleiotropic actions in oxidative and inflammatory system and neurotransmitters [33].

## 4. Therapeutic Strategies and the Gut–Brain Axis: Current Approaches, Emerging Focus, and Challenges

### 4.1. Current Therapeutic Approaches and Challenges

Although decades of research have been conducted, dementia does not have curative treatment. Treatment approaches have been oriented to symptom control and delaying the progression of the disease and have not aimed at reversing the neurodegenerative processes themselves [34]. This is indicative of the complexity of the pathogenesis of dementia as follows: the presence of amyloid-beta, hyperphosphorylation of tau, oxidative stress, neuroinflammation, and the dysfunction of the gut–brain axis. Multi-modal approaches are essential as single-target therapies have shown to be inadequate [35].

Cholinesterase inhibitors (donepezil, rivastigmine, and galantamine) and the NMDA receptor antagonist memantine have served as the foundation of pharmacological treatment of the Alzheimer’s disease. The cholinesterase inhibitors have the effect of inhibiting acetylcholinesterase thus elevating the levels of acetylcholine in the synaptic cleft to enhance neurotransmission and cognitive functioning [36]. The most commonly prescribed is donepezil, which has moderate effectiveness in mild to moderate AD, and rivastigmine and galantamine have the same advantages with minor differences in tolerability [37]. Memantine is taken in moderate to severe stages and functions to control glutamate signaling to inhibit excitotoxic neuronal death [38]. However, these medications have only temporary and slight benefits. They fail to stop neurodegeneration and have adverse effects like nausea, vomiting, bradycardia, and dizziness [39]. Also, they have low systemic half-lives and poor penetration across the blood–brain barrier, which hamper their long-term effectiveness [40]. This pharmacological limit has led to the investigation of adjunctive and alternative medicine.

Non-pharmacological interventions are useful as supportive treatment in enhancing quality of life and preventive cognitive impairment. The dietary interventions, specifically, the Mediterranean and dietary approaches to stop hypertension (DASHs), have shown some improvements in memory, attention, and executive function [41]. Exercise leads to better neurogenesis and synaptic plasticity, and meditation and computer-based cognitive training led to better attention and processing speed [42]. The value of lifestyle factors with regard to dementia management has also been connected with better cognitive outcomes, which is associated with sleep optimization. These solutions are appealing since they are not risky and are widely available. Nevertheless, they tend to be relatively small in their effectiveness and rely on adherence which makes them ineffective as a single intervention. They should be considered as complementary measures that might be synergetic with pharmacological or nutraceutical measures.

### 4.2. Emerging Focus: The Gut–Brain Axis, Challenges and Limitations

A particularly promising frontier is the gut–brain axis. Gut dysbiosis interferes with intestinal and blood–brain barrier integrity, increases endotoxin (LPS) levels, and activates microglia, which leads to neuroinflammation and cognitive decline [33]. Probiotics, prebiotics, and those high in polyphenols have demonstrated potential to restore gut microbiota homeostasis, mitigate oxidative stress and alter systemic inflammation [43,44]. This body of work indicates that peripheral immune activation is a potential therapeutic target to indirectly reduce central neurodegeneration.

Although these advances have been made, there are a number of challenges. The pharmacological therapy is still restricted due to side effects and absence of disease-modifying effects. Although they have their advantages, non-pharmacological interventions have limitations due to adherence and small effect sizes. The gut–brain axis therapies are not yet fully developed and have a shortage of clinical validation. Furthermore, the differences in patient response point to the necessity to consider individualized strategies that would combine genetic, lifestyle, and environmental variables. The therapeutic scenery is therefore one that shows advancement and disappointment. Although the symptoms have been managed, the lack of curative or disease-modifying therapies highlights the need to consider complementary approaches. Herbal medicines and especially with multi-target action have become the promising choices to be used to occupy this gap. This preconditions the analysis of BM, a traditional herb with a growing scientific confirmation [43,44].

## 5. *Bacopa monnieri* in Traditional and Modern Medicine: Phytochemical Constituents, Mechanism of Action, and Limitations in Application

*Bacopa monnieri* (BM) or brahmi, also known as water hyssop, is a centuries-old Ayurvedic medicine that is considered a medhya rasayana, or intellect and memory rejuvenator [45]. It was traditionally prescribed to boost intelligence, life span, and spiritual clarity and it is a major ingredient in the formulations aimed at anxiety, inadequate cognition, and lack of concentration [46]. The ease with which it spread into Asia, Australia and the United States enabled its use in a variety of ethnobotanical contexts though its most culturally and medically significant presence is found in South Asia, especially in India and Thailand [47]. This historical confirmation offers a basis to contemporary scientific research. In contrast to most herbal remedies whose traditional assertions have not been supported by empirical research, BM has been shown to be a cognitive enhancer with preclinical and clinical studies, showing the link between ancient practice and modern neuroscience [47].

**Figure 3:**
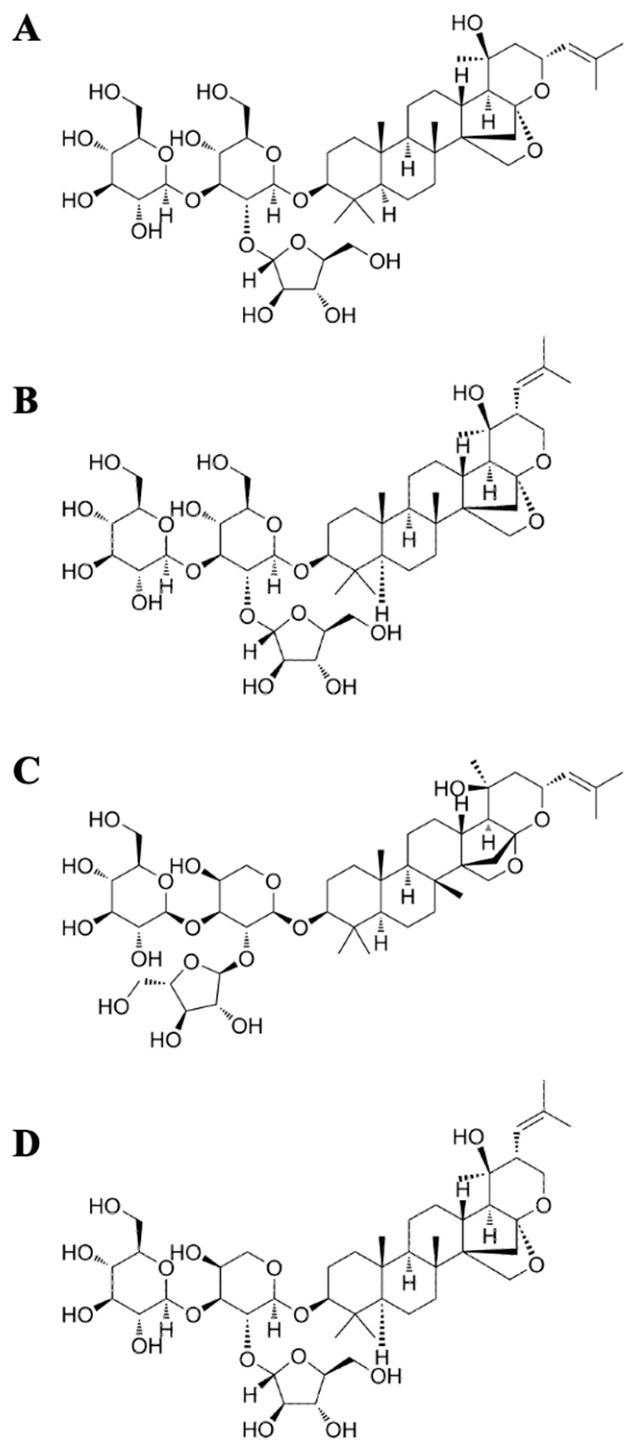
The structure of bacoside A mixtures in *Bacopa monnieri*. (**A**) bacoside A_3_, (**B**) bacopaside II, (**C**) bacopaside X, and (**D**) bacopasaponin C (Adapted from MedChemExpress, 2026).

The pharmacological activity of BM is supported by a wide range of bioactive compounds such as saponins, alkaloids, steroids, and flavonoids [48]. These include the neuroactive fraction that is mostly represented by the saponin glycosides, which are triterpenoids and are collectively called bacosides (Figure 3; Table 2). The well-characterized sub-fraction bacoside A, which is a mixture of bacoside A3, bacopaside II, bacopaside X, and bacopasaponin C, has been most extensively investigated concerning its neuroprotective and memory-enhancing effects [49]. They can exert antioxidant, anti-inflammatory, anti-amyloidogenic, anti-tau, anti-apoptotic, and neurogenic actions and affect several molecular targets relevant to AD, which makes a comprehensive treatment of the multifactorial pathology of AD possible [9].

In terms of signal transmission, at the neuroinflammatory level, the anti-inflammatory action of bacosides is mainly due to the inhibition of the NF-κB and MAPK cascades. Glial inflammatory activation in activated microglia and astrocytes results from activation of the NF-κB signaling cascade and MAPKs and contributes to neurodegenerative diseases when dysregulated, leading to chronic release of pro-inflammatory cytokines such as IL-1β, IL-6, and TNF-α, which trigger synapse loss, cognitive dysfunction, and mitochondrial damage. These cascades have been demonstrated to be reduced by BM extract, which results in less glial hyperactivation and subsequent cytokine production. Human evidence of anti-inflammatory effects of BM has been shown in a clinical trial over 3 months in cognitively healthy subjects, which showed a measurable reduction in NF-κB phosphorylation after BM supplementation compared to the control group. The antioxidant action of bacoside is mediated by the activation of the Nrf2/Keap1 pathway. BM has been shown to exert its effects on oxidative stress through activation of nuclear factor erythroid 2-related factor 2 (Nrf2) by Kelch-like ECH-Associated Protein (Keap1) expression, which will lead to up-regulation of Glutathione synthesis and other antioxidant enzymes of phase II like heme oxygenase-1 (HO-1) and glutamate cysteine ligase catalytic subunit (GCLC), which reinforces the cells’ ability to fight against reactive oxygen and nitrogen species thought to play a role in AD neurodegeneration [50].

Regarding amyloid pathology, BM phytochemicals have been shown to have inhibitory activity towards BACE1, the rate-limiting enzyme in amyloidogenic processing of APP. Amyloid-beta peptides are generated by sequential cleavage of APP by BACE1 and γ-secretase, and BACE1 is a key and desired therapeutic target for AD. Bacosides have been shown to possess anti-amyloid and antioxidant activities that involve interference with aggregation of Aβ and reduction in oxidative stress-induced neurotoxicity, with molecular docking studies showing binding energies of −8.9 kcal/mol with the BACE1 active site, hydrogen bonding with tyrosine residue at position 71 (Tyr71), tyrosine residue at position 198 (Tyr198), and threonine residue at position 231 (Thr231) residues. This structural interaction supports their mechanism of action at the enzyme level as anti-amyloidogenic agents [51]. Secondly, the impact of BM on tau pathology, a downstream marker of AD that directly correlates with the severity of neurodegeneration, is also important. BM treatment in Neuro2a cells was able to decrease the phospho-tau load and phosphorylation of glycogen synthase kinase-3 beta (GSK-3β), a major tau kinase, and to increase Nrf2 levels and reduce ROS and caspase-3 activity [52,53]. In a rodent model of AD, induced by amyloid beta-42 (Aβ42), BM activity reduced amyloid plaques in the hippocampus and normalized the Aβ42-induced increase in phospho-tau and total tau expression, and the anti-Alzheimer effect of BM was also shown to be related to its effect on mitochondrial dysfunction and modulation of GSK3β-mediated wingless-related integration site/beta-catenin (Wnt/β-catenin) signaling pathway. This pathway is especially pertinent because both tau hyperphosphorylation and inhibition of neuro-protective Wnt signaling are mediated by GSK-3β hyper-activation in AD, and BM’s ability to normalize this axis is a mechanistically important intervention [52,53]. The bacosides act at the synaptic and neurotransmitter level, on both the cholinergic and the serotonergic system. BM inhibits acetylcholinesterase (AChE), which increases the amount of acetylcholine available in the synapse, thereby opposing the cholinergic deficiency in the early stages of cognitive impairment associated with AD. BM works mainly through antioxidant activity or by changing the activity of neurotransmitters such as serotonin (5-Hydroxytryptamine, 5-HT), which fine-tune neural plasticity as a substrate for memory formation, and dopamine, acetylcholine, and gamma-aminobutyric acid (GABA). BM supplementation in an AD rat model resulted in a decrease in the activity of cholinesterase, restoration of Bcl-2-associated X protein/B-cell lymphoma 2 (Bax/Bcl-2) balance, upregulation of the expression of neurotrophic factor, and prevention of neurodegeneration, as confirmed by quantification of Nissl-positive neurons in the hippocampus. These all act to safeguard the integrity of synapses and promote mechanisms that facilitate long-term potentiation, which is important for learning and memory [54,55].

Interspecific interaction between the bacosides and complementary phytochemicals present in BM, such as flavonoids, phenolics, and alkaloids, adds to the pharmacological profile of the bacosides. Flavonoids like quercetin have been found to interact with inducible nitric oxide (iNOS), cyclooxygenase-2 (COX-2), matrix metalloproteinase-9 (MMP9), and signal transducer and activator of transcription 3 (STAT3) to further inhibit the pathways of neuroinflammation [56]. This phytochemical complement makes BM a truly multi-target agent that can simultaneously target the NF-κB/MAPK inflammatory axis, NLRP3-dependent oxidative priming, BACE1-mediated amyloidogenesis, GSK-3β-mediated tau pathology and cholinergic synaptic defects, well suited for the multifactorial nature of AD pathogenesis.

This mechanistic information is being supported with clinical data. Verbal learning and memory acquisition, as well as delayed recall, were improved in healthy elderly subjects with BM supplementation, though gastrointestinal side effects were observed [57]. In AD patients, standardized BM extract showed higher improvement in MMSE scores in the domains of orientation, attention, and language and better improvements in quality of life and irritability and insomnia [58]. Polyherbal preparation with BM has also shown cognition enhancement activity along with synergistic neuroprotection with other neuro-protective botanicals [59].

This encouraging evidence comes despite some very significant challenges in terms of the clinical translation of BM. Bioavailability and therapeutic consistency are restricted by the poor solubility in aqueous media, bitter taste, and heat, acid, and moisture sensitivity of bacosides [11]. The pharmacokinetic limitations highlight the importance of implementing advanced formulation strategies like micro-/nanoencapsulation, lipid-based formulations, and cyclodextrin complexation to ensure the integrity of phytochemicals, promote gastrointestinal absorption, and ensure their safe delivery in the central nervous system. If it is not for these advances, the pharmacokinetic constraints of BM will remain limiting to making the impressive mechanistic properties into clinically relevant results.

**Table 2:** Neuroprotective effects of bacoside A and mixtures.

Active Constituents	In Vitro	In Vivo	Clinical Trial	References
Bacoside A (Fresh whole plant: 1 g/100 g) (Dried whole plant: 45 g/100 g) (Leaf: 4.35–10.50% *w*/*w*)	N2a cells (0.4 mg/mL) SH-SY5Y cells (50 μM)	C57BL/6 mice (10 and 20 mg/kg) Swiss mice (5 mg/kg) Swiss mice (5 mg/kg) Swiss mice (5 mg/kg) Swiss mice, Wistar rat (5 mg/kg) Wistar neonatal rats (50 mg/kg) Wistar rats (10 mg/kg) Wistar rats (10 mg/kg) Wistar rats (150 mg/kg)	N/A	[60] [49] [61] [50] [62] [62] [63] [64] [65]
Bacoside A3 (Aerial parts of plant: 1.13% *w*/*w*) (19.5% *w*/*w* of bacoside A) Bacopaside Il (26.9% *w*/*w* of bacoside A) Bacopaside X (32.3% *w*/*w* of bacoside A) Bacopasaponin C (17.2% *w*/*w* of bacoside A)	U87MG cells (0.5–5 μM) N/A N/A N/A	N/A N/A Swiss mice (20 mg/kg) N/A	N/A N/A N/A N/A	[66] [56] [56] [67]

## 6. Neuroprotective Effect of *Bacopa monnieri*: Evidence from Preclinical and Clinical Studies

### 6.1. Preclinical Evidence

The neuroprotective action of BM has been elucidated with the help of rodent models (Table 3). These models recapitulate features of Alzheimer pathology, such as oxidative stress, neuroinflammation, and neurotransmitter dysfunction and enable researchers to study the mechanisms of action of BM. Research has revealed that BM improves the learning, memory, and attention of rodents, and the effects are correlated with the ability to modulate the cholinergic signaling, decrease the oxidative stress, and inhibit the pro-inflammatory cytokines [68,69]. As an example, bacoside A was investigated to prevent amyloid-beta toxicity, limit lipid peroxidation and up-regulate antioxidant enzymes in the brain of the mice and rats [65,66]. Notably, BM has an impact on cognitive performance and structural and biochemical markers. There are rodent studies that report increased dendritic branching, increased synaptic density, and decreased neuronal apoptosis with the use of BM [63,64]. These results indicate that BM does not only enhance functional recovery, but also neurogenesis and synaptic resilience, which are essential to long-term cognitive well-being. However, preclinical research does not unconditionally have no limitations. The majority of experiments are done on male animals and sex-specific effects have not been explored. Dosage and administration are very wide, making it difficult to compare it across studies. Additionally, although rodent models are informative, their applicability in translating human dementia has been limited by the species disparities in metabolism and brain complexity.

### 6.2. Clinical Evidence

The literature on the clinical benefits of BM has been limited, except that human trials have given more evidence of cognitive benefits of BM (Table 4). BM extract (300 mg/day) supplement over 12 weeks significantly enhanced verbal/learning, memory acquisition, and delayed recall relative to placebo in healthy elderly individuals, but with gastrointestinal side effects of abdominal cramps and nausea [57]. Standardized BM extract (300 mg two times a day during six months) was also found to positively affect the MMSE scores, especially in the areas of orientation, attention, and language, as well as quality of life and irritability and insomnia in patients with newly diagnosed AD [58]. BM has also been shown to be promising in polyherbal formulations. A randomized controlled trial of BM with *Hippophae rhamnoides* and *Dioscorea bulbifera* showed positive effects on cognitive tests like the Digit Symbol Substitution Test and word recall, decreased inflammation and oxidative stress [59]. These results indicate that synergistic effects of BM can be used with other neuroprotective herbs, in line with Ayurvedic concepts of multi-herbal preparations. Although these are promising outcomes, clinical evidence is still rudimentary. It is difficult to replicate the results due to small sample sizes, relatively short trials, and variability in the extract standardization. Also, although cognitive gains are regularly identified, there is a lack of long-term results including disease progression and neurodegeneration. The safety profiles are mostly favorable with gastrointestinal side effects pointing to the need to improve formulations to increase tolerability.

Combined, preclinical and clinical data indicate BM as a multi-target and cognitive enhancer. Its neurotransmitter-modulating, oxidative stress-reducing, and neuroinflammatory-inhibitory properties are in line with the multifactorial pathophysiology of dementia. However, the strength of existing evidence is limited by methodological shortcomings, such as small sample sizes, uneven dosing, and a non-standardized extract. The lack of translations to human dementia relative to rodent models also underscores the need to have large-scale clinical trials that are rigorous. The potential of BM is its pleiotropic action that can be used to supplement traditional therapy and lifestyle intervention. However, to achieve this potential, there is a need to overcome pharmacokinetic issues, especially low bioavailability and instability. This has led to a surge of interest in novel delivery methods like microencapsulation which could lead to a better clinical effect of BM by increasing stability, solubility and localized release.

## 7. *Bacopa monnieri* Challenges and Innovations

Although BM has good pharmacological potential, the use of this herb has serious clinical problems. The main weakness is that the active constituents, especially bacosides of this product, are not well bioavailable. These compounds are only slightly soluble in water, bitter, heat and acid labile and worsen their therapeutic effectiveness [11]. Accordingly, although BM has potent effects in vitro and in animal models, pharmacokinetic factors hamper its clinical use. The discrepancy between the potential of laboratories and patient outcomes underscores the need for emerging delivery systems.

Microencapsulation has come up as a potential solution to overcome these setbacks. The technology enhances bioactive compounds stability, adversity to bad taste, solubility and controlled release and target delivery; it involves encircling bioactive compounds within microcapsules, which are composed of core and wall layers [12,139]. They have used spray-drying, coacervation, and liposomal encapsulation to make sure that sensitive molecules are not destroyed by environmental factors and increase absorption in the intestine [139]. Microencapsulation has a number of benefits to BM. It enhances biological activity, has a more stable neuroprotective effect on the living organism, and improves the effectiveness of treatment by enhancing intestinal absorption [51,140,141]. The new technology is an immediate reaction to the pharmacokinetic complications which have limited BM to clinical applications, rendering it a promising candidate in functional foods and nutraceuticals to aid in cognitive health. The concept of microencapsulation is a breakthrough, but more research is needed to fine-tune the formulations and make them effective in clinical practice. It is not compared with the microencapsulated form and there is a lack of comparative studies between the BM extract and the questions about the relative effectiveness are not answered. Safety and tolerability are also to be rated critically on long-term basis, particularly in the elderly populations where dementia is very common.

Other innovative techniques are also being taken into account in addition to microencapsulation. BM can also be enhanced using the delivery system of nanoparticles, phytochemical standardization and polyherbal preparations to enhance its therapeutic potential. Interaction with conventional pharmacotherapy and lifestyle modifications can be synergistic because a growing pattern is in the direction of multi-modal management of dementia. The following issues of BM can be seen as typical of the general challenges of the field of research on herbal medicine: promising bioactivity being undermined by absence of pharmacokinetics and untrustworthy formulations. The new technologies such as microencapsulation are feasible solutions, but they have to be highly validated and standardized to succeed. Addressing such concerns will be critical towards transforming the historical reputation and clinical potential of BM into plausible clinical results. In this way, BM can become a source of complementary measures to be taken regarding dementia and the relation between old knowledge and the new one [51,140,141].

## 8. Conclusions

One of the most daunting health issues in the world is dementia and especially AD. Its multifactorial nature that includes amyloid-beta aggregation, tau hyperphosphorylation, oxidative stress, neuroinflammation, and gut–brain axis dysregulation has been challenging to address using a single-actor response. There are some moderate symptomatic effects and non-pharmacological therapy provided by existing pharmacological therapy but not enough to prevent the disease progression. The field of therapy has resulted in a greater interest in complementary therapies and especially pleiotropic herbal medicines. Among them, BM is one of the most promising candidates. Having centuries of Ayurvedic use and increasing evidence of cognitive-enhancing and neuroprotective effects in preclinical and clinical research, BM has demonstrated both cognitive-enhancing and neuroprotective effects. Its active ingredients, in particular, bacosides, control neurotransmission, reducing oxidative stress and preventing neuroinflammation, which is also accompanied by the multifactorial character of the dementia pathology. Clinical trials, which have demonstrated improvement in memory, attention and quality of life, are limited to supporting the concept of BM as a complementary therapy.

Nevertheless, there are critical issues left. BM is poorly bioavailable and unstable in environmental conditions and variability in formulations limits its clinical use. Limitations in methodology include sample sizes, trial duration, and lack of standardized extracts, which further weaken the evidence base. Solving these problems is essential in transforming BM into a promising adjunct into a trustworthy treatment method. Examples of such innovations include microencapsulation, which improves stability, solubility, and directed delivery. Further studies ought to focus on comparative research on BM extract and microencapsulated formulations, safety assessment over the long term and combination with traditional therapies. The understanding of the role of BM in the regulation of the gut–brain axis and the opportunities of its application in polyherbal or nutraceutical preparations can also increase the therapeutic value of this substance.

Overall, BM serves as an example of how herbal medicines can be used to supplement traditional treatments of dementia. Its multi-targeted processes are in line with the complexity of neurodegeneration, and its classical validation supports its cultural and medical relevance. To realize this potential, a stringent scientific investigation, formulation strategy, and integrative strategies that incorporate ancient knowledge with modern biomedical science are necessary. Provided that these issues are addressed, BM may contribute significantly to the reduction in the burden of dementia and promotion of the cognitive health of older generations.

## Figures and Tables

**Table 1:** The top 10 countries with the highest cost of dementia and the estimated number of people in the Asia–Pacific region.

Country	Estimated Costs (US$)	Estimated Number of People with Dementia		
		2015	2030	2050
Japan	93,240,000	3,014,000	4,421,000	5,214,000
China	44,619,000	10,590,000	18,116,000	32,184,000
Australia	12,892,000	328,000	520,000	864,000
Korea	8,676,000	462,000	974,000	2,113,000
Taiwan	6,990,000	260,000	461,000	840,000
India	4,620,000	4,031,000	6,743,000	12,542,000
Hong Kong	3,227,000	115,000	212,000	436,000
Thailand	1,810,000	600,000	1,117,000	2,077,000
Indonesia	1,777,000	1,033,000	1,894,000	3,979,000
Singapore	1,664,000	45,000	103,000	241,000

**Table 3:** Animal model induced for cognitive and memory impairment associated with Alzheimer’s disease.

Model	Species	Age	Gender	Biomarker	Dose	References
Streptozotocin	Sprague–Dawley rats	5 months	Female	Amyloid plaques, tauopathy, gliosis, atrophy of parenchyma, neurochemical alterations, oxidative stress, neuroinflammation	1.5 mg/kg (i.c.v)	[70]
	Wistar albino rats	3–4 months	Male		3 mg/kg (i.c.v)	[71]
	Sprague–Dawley rats	1 years	Male		0.25 mg/μL (i.c.v)	[72]
Amyloid-β	Fisher rats	18–20 weeks	Male	Amyloid plaques, tau phosphorylation, cholinergic deficits, neuroinflammation, glutaminergic alterations	20 μg/3 days (i.c.v)	[73]
	Long–Evans rats	2–3 months	Male		4 μg/μL (i.c.v)	[74]
Scopolamine	C57BL/6J mice	7–12 weeks	Female	Tau pathology, cholinergic impairment, monoamine alterations, neuroinflammation	10 mg/kg (i.p)	[75]
	Albino rats	4 months	Male		1 mg/kg (i.p)	[76]
	Swiss mice	10–12 weeks	Male/ female		1 mg/kg (i.p)	[77]
Ethylcholine aziridinium ion	Wistar–Imamichi rats	7–8 weeks	Male	Cholinergic deficits, astrogliosis	2 nmol/2 μL (i.c.)	[78]
	Sprague–Dawley rats	5 weeks	Male		6 nmol/6 μL (i.c.v)	[79]
Alcohol	Wistar rats	2–3 months	Male	Deficits in spatial memory, oxidative stress, neuroinflammation, neuronal loss	20% *v*/*v*; 4 g/kg (p.o.)	[80]
	Wistar rats	2–3 months	Male		4 g/kg (p.o.)	[81]
	Sprague–Dawley rats	2 months	Male		20% *v*/*v*; 4 g/kg (p.o.)	[82]
	Long–Evans rats and Sprague–Dawley rats	2–3 months	Male		20% *v*/*v*; 1 or 4 g/kg (p.o.)	[83]
Colchicine	Wistar rats	8–10 weeks	Male	Amyloid plaques, neuroinflammation, cognitive deficits, NMDA activation, neurodegeneration	15 μg/5 μL (i.c.v)	[84]
	Adult albino rat	6–8 weeks	Male		15 μg/5 μL (i.c.v)	[85]
	Adult albino rat	6–8 weeks	Male		7.5 μg/10 μL (i.c.v)	[86]
^192^IgG-saporin	Sprague–Dawley rats	2 months	Male	Cholinergic deficits, cognitive deficits, synaptic changes, glial changes	100, 237.5, or 375 ng/0.3 μL (i.c.v)	[87]
	Sprague–Dawley rats	2 months	Male		8 μg/0.3 μL (i.c.v)	[88]
	Sprague–Dawley rats	2 months	Male		4 μg/0.3 μL (i.c.v)	[89]
Aluminum	Wistar albino rats	6–12 months	Male/ female	Amyloid plaques, tangles, neurotransmitter alterations	4.2 mg/kg (i.p.)	[90]
	Sprague–Dawley rats	15 weeks	Male		100 mg/kg (p.o.)	[91]
	Wistar rats	6–12 months	Male		10 mg/kg, (i.p.)	[92]
	Sprague–Dawley rats	12 weeks	Male		100 mg/kg (p.o.)	[93]
Methionine	Wistar rats	3 months	Male	Cholinergic deficits, endothelial dysfunction, neuroinflammation, gliosis, cognitive deficits, oxidative stress	1.7 g/kg (p.o.)	[94]
	Wistar albino rats	3 months	Male		1.7 g/kg (p.o.)	[95]
Okadaic acid	Sprague–Dawley rats	2 months	Male	Tau phosphorylation, amyloid-β plaques, oxidative stress, cognitive deficits	200 ng/5 μL (i.c.v)	[96]
	Sprague–Dawley rats	3 months	Female		7 or 70 ng/day (i.c.v)	[97]
Ibotenic acid	Sprague–Dawley rats	2–3 months	Male	Oxidative stress, apoptosis, cognitive deficits, excitotoxicity	2 μg/μL (i.c.v)	[98]
Clonidine	Wistar rats	2–3 months	Male	Cognitive deficits	0.1 mg/kg (i.p.)	[99]
	Wistar rats	2–3 months	Male		0.1 or 0.2 mg/kg (i.p.)	[100]
Clozapine	Sprague–Dawley rats	2–3 months	Female	Cognitive deficits	1.25 and 2.5 mg/kg (s.c)	[101]
	Sprague–Dawley rats	2–3 months	Female		1.25 and 2.5 mg/kg (s.c)	[102]
Lignocaine	Wistar rats	2–3 months	Male	Cognitive deficits	4% *w*/*v*; 0.5 μL (i.c.v)	[103]
	Wistar rats	2–3 months	Male		2% *w*/*v*; 1 μL (i.c.v)	[104]
Cycloheximide	C57BL/6J mice	8–10 weeks	Male	Cholinergic deficits, cognitive disturbances, neurotransmitter alterations	3.5, 7, 15, 30, 75, 150 mg/kg (s.c.)	[105]
	Sprague–Dawley rats	8–10 weeks	Male		1 mg/kg (s.c.)	[106]
Phenytoin	Wistar rats	2–3 months	Male	Oxidative stress, cognitive deficits	75 mg/kg (p.o.)	[107]
	Wistar rats	2–3 months	Male		5, 12.5, 25, 50, or 75 mg/kg (i.p.)	[108]
D-Galactose	Swiss albino mice	6–8 weeks	Male	Amyloid-β, neuroinflammation, oxidative stress, cognitive deficits	150 mg/kg (s.c.)	[109]
	C57BL/6J mice	6–8 weeks	Male		150 mg/kg (i.p.)	[110]
	C57BL/6J mice	12 weeks	Male		100 mg/kg (s.c.)	[111]
	C57BL/6J mice	6–8 weeks	Male		150 mg/kg (s.c.)	[112]
	C57BL/6J mice	19–20 weeks	Male		100 mg/kg (s.c.)	[113]
	Wistar rats	2–3 months	Male		150 mg/kg (s.c.)	[114]
	Sprague–Dawley rats	4 months	Male		160 mg/kg (s.c.)	[115]
Dizocilpine	Wistar rats	8–10 weeks	Male	Cognitive deficits, motor disturbances	1 mg/kg (i.p)	[116]
Diazepam	Swiss albino mice	Young (3–4 months) and aged (12–15 months)	Male	Cognitive deficits	1 mg/kg (i.p.)	[117]
	Swiss albino mice	3 months	Male		1 mg/kg (i.p.)	[118]
Lipopolysaccharide	Sprague–Dawley rats	3 months	Male	Neuroinflammation, oxidative stress, neurochemical alterations, amyloid plaques, tauopathy, gliosis	0.25 μg/h (i.c.v.)	[77]
	Long–Evans rats and Sprague–Dawley rats	35 days 10–12 weeks	Male/ female		0.125, 0.25 and 0.50 mg/kg (i.p.)	[119]
	Wistar rats	13–14 weeks	Male		0.5–5 μg/2 μL (i.c.v.)	[120]
	Wistar rats	8–12 weeks	Male		100 and 250 μg/kg (i.p.)	[121,122]
	Wistar rats	8–10 weeks	Male		5 mg/kg (i.p.)	[123]
	Wistar rats	8 weeks	Male		1 mg/kg (i.p.)	[124,125]
	C57BL/6J mice	11–12 weeks	Male		500 and 750 μg/kg (i.p) 12 μg/3 μL (i.c.v)	[28]
	Swiss albino mice	6–8 weeks	Male		0.25 mg/kg (i.p.)	[126]

**Table 4:** Neuroprotective effects of *Bacopa monnieri* associated with cognitive aspect and memory in clinical trials.

Population	Intervention	Outcome	Result	Side Effect	References
Healthy adult (18–44) n = 24	BM extract (320 and 640 mg/daily) /placebo for 2 h	Cognitive, mood, and cortisol level	↑ cognitive ↑ positive mood ↓ cortisol level	N/A	[127]
Healthy adult (18–56) n = 24	BM extract (320 and 640 mg/daily)/placebo for 2 h	Cognitive, stress, fatigue, and blood pressure	↑ cognitive	Gastrointestinal discomfort	[128]
Healthy adult (18–60) n = 38	BM extract (300 mg/daily) /placebo for 2 h	Neuropsychological	No difference in cognitive	N/A	[129]
Healthy adult (18–60) n = 107	BM extract (300 mg/daily) /placebo for 90 days	Cognitive	↑ cognitive	Diarrhea	[130]
Healthy adult (18–60) n = 46	BM extract (300 mg/daily) /placebo for 12 weeks	Neuropsychological, anxiety	↑ cognitive ↑ memory ↓ anxiety	Nausea, dry mouth, fatigue	[131]
Healthy adult (19–22) n = 60	BM extract (300 mg/daily) /placebo for 6 weeks	Neuropsychological, biochemical	↑ cognitive ↑ memory ↑ serum calcium level	N/A	[132]
Healthy adult (35–60) n = 72	BM extract (450 mg/daily) /placebo for 12 weeks	Cognitive, anxiety	No difference in cognitive	N/A	[133]
Healthy older (≥55) n = 40	BM extract (250 mg/daily) /placebo for 16 weeks	Cognitive, memory	↑ cognitive ↑ memory ↑ mental control	Rash	[134]
Healthy older (≥55) n = 98	BM extract (300 mg/daily) /placebo for 12 weeks	Neuropsychological	↑ memory	Increase stool frequency, abdominal cramps, nausea	[59]
Healthy older (≥55) n = 28	BM extract (320 mg/daily) /placebo for 12 weeks	Cognitive, psychological, biochemical	↑ cognitive	N/A	[135]
Healthy older (≥60) n = 60	BM extract (300 and 600 mg/daily) /placebo for 12 weeks	Cognitive, memory, biochemical	↑ cognitive ↑ memory ↑ attention ↓ AChE	N/A	[136]
Healthy older (≥65) n = 54	BM extract (300 mg/daily) /placebo for 12 weeks	Cognitive, anxiety, depression, heart rate	↑ cognitive ↓ anxiety ↓ depression ↓ heart rate	Flu-like symptoms, digestive problems	[137]
AD and MCI-AD (≥50) n = 34	BM extract (300 mg/daily) /donepezil (10 mg/daily) for 12 months	Neuropsychological	No difference between BM and donepezil	Nausea, diarrhea, asthenia, arthralgia, headache, dizziness, anxiety, restlessness, insomnia, crying	[138]

NOTE: ↑ means increase, ↓ means decrease; n—number of animals.

## Data Availability

No new data were created or analyzed in this study. Data sharing is not applicable to this article.

## Abbreviations

The following abbreviations are used in this manuscript:

5-HT	5-Hydroxytryptamine
ABS	Amyloid-beta-species
AChE	Acetylcholinesterase
AD	Alzheimer’s disease
ADI	Alzheimer’s Disease International
AP-1	Activator protein 1
APP	Amyloid precursor protein
ASC	Apoptosis-associated speck-like protein containing a caspase recruitment domain
ATP	Adenosine triphosphate
AVLT	Auditory verbal learning test
Aβ	Amyloid beta
Aβ42	Amyloid beta-42
BACE1	Beta-site amyloid precursor protein cleaving enzyme 1
Bax	Bcl-2-associated X protein
Bcl-2	B-cell lymphoma 2
BM	*Bacopa monnieri*
C1q	Complement component 1q
CCL2	C-C motif chemokine ligand 2
CD11b	Cluster of differentiation 11b
CD36	Cluster of differentiation 36
COX-2	Cyclooxygenase-2
DALYs	Disability-adjusted life years
DAMPs	Damage-associated molecular patterns
DASHs	Dietary approaches to stop hypertension
GABA	Gamma-aminobutyric acid
GCLC	Glutamate cysteine ligase catalytic subunit
GSK-3β	Glycogen synthase kinase-3 beta
HO-1	Heme oxygenase-1
IL-1	Interleukin-1
IL-12	Interleukin-12
IL-18	Interleukin-18
IL-1α	Interleukin-1-alpha
IL-1β	Interleukin-1-beta
IL-2	Interleukin-2
IL-23	Interleukin-23
IL-6	Interleukin-6
iNOS	Inducible nitric oxide synthase
JNK	c-Jun N-terminal kinase
Keap1	Kelch-like ECH-associated protein
LPSs	Lipopolysaccharides
MAPK	Mitogen-activated protein kinase
MHC-II	Major histocompatibility complex class II
MMP9	Matrix metalloproteinase-9
MMSE	Mini-mental state examination
MyD-88	Myeloid differentiation primary response protein 88
NF-kB	Nuclear factor kappa-light-chain-enhancer of activated B cells
NFTs	Neurofibrillary tangles
NLRP3	NOD-like receptor family pyrin domain-containing 3
NMDA	N-methyl-D-aspartate
NO	Nitric oxide
Nrf2	Nuclear factor erythroid 2-related factor 2
_P_40	Nuclear factor kappa B subunit 40
_P_53	Nuclear factor kappa B subunit 53
_P_65	Nuclear factor kappa B subunit 65
PRRs	Pattern recognition receptors
RAGEs	Receptor for advanced glycation end-products
ROS	Reactive oxygen species
STAT3	Signal transducer and activator of transcription 3
Thr231	Threonine residue at position 231
TLR2	Toll-like receptor 2
TLR4	Toll-like receptor 4
TNFR1	Tumor necrosis factor receptor 1
TNF-α	Tumor necrosis factor alpha
Tyr198	Tyrosine residue at position 198
Tyr71	Tyrosine residue at position 71
WHO	World Health Organization
Wnt/β-catenin	Wingless-related integration site/beta-catenin signaling pathway
